# Near-Infrared-II Cyanine/Polymethine Dyes, Current State and Perspective

**DOI:** 10.3389/fchem.2021.718709

**Published:** 2021-07-29

**Authors:** Yijing Du, Xiangping Liu, Shoujun Zhu

**Affiliations:** ^1^ State Key Laboratory of Supramolecular Structure and Materials, College of Chemistry, Jilin University, Changchun, China; ^2^ Joint Laboratory of Opto-Functional Theranostics in Medicine and Chemistry, The First Hospital of Jilin University, Changchun, China

**Keywords:** near-infrared-II, cyanine/polymethine dyes, multiplexed channels, molecular imaging, pharmacokinetics

## Abstract

The development of near-infrared-II (NIR-II) fluorescence imaging has implemented real-time detection of biological cells, tissues and body, monitoring the disease processes and even enabling the direct conduct of surgical procedures. NIR-II fluorescence imaging provides better imaging contrast and penetration depth, benefiting from the reducing photon scattering, light absorption and autofluorescence. The majority of current NIR-II fluorophores suffer from uncontrollable emission wavelength and low quantum yields issues, impeding the clinical translation of NIR-II bioimaging. By lengthening the polymethine chain, tailoring heterocyclic modification and conjugating electron-donating groups, cyanine dyes have been proved to be ideal NIR-II fluorophores with both tunable emission and brightness. However, a simpler and faster method for synthesizing NIR-II dyes with longer wavelengths and better stability still needs to be explored. This minireview will outline the recent progress of cyanine dyes with NIR-II emission, particularly emphasizing their pharmacokinetic enhancement and potential clinical translation.

## Introduction

Near infrared fluorescence imaging technology is widely used in tracking biological processes and disease diagnosis due to its non-invasive, real-time and multi-dimensional monitoring characteristics. Indocyanine green (ICG) and methylene blue (MB) has been approved by the US Food and Drug Administration (FDA) for clinical using, such as angiography or directing cancer surgery (Chen et al., 2019). Contrast to the NIR-I (700–1,000 nm) region, the NIR-II (1,000–1,700 nm) region has higher tissue penetration depth and imaging contrast for mammalian bioimaging (Frangioni, 2003), resulting from the reducing photon scattering, light absorption and tissue autofluorescence (Hong et al., 2017). Initially, some inorganic materials were developed for NIR-II imaging, given the examples like carbon nanotubes (Welsher et al., 2009), quantum dots (Bruns et al., 2017; Li et al., 2015), rare Earth doped nanoparticles (Fan et al., 2018; Naczynski et al., 2013; Zhong et al., 2017) or gold nanocluster (Liu et al., 2019; Zhu et al., 2018a). These materials usually have relatively high quantum yield and tunable emission wavelengths, which is particularly advantageous for vessel (Hong et al., 2014), liver or kidney (Loynachan et al., 2019) imaging. However, the long-term retention and unconfirmed biotoxicity limit their clinical translation (Zhang et al., 2013).

Researchers then refocused on organic dyes with good biocompatibility and fast excretion post-imaging. A variety of NIR-II dyes have been synthesized, such as donor-acceptor-donor (D-A-D) (Antaris et al., 2016) and cyanine fluorophores. D-A-D molecules have relatively low extinction coefficient and moderate quantum yield in nonpolar organic solvent, while they are easily quenched by water and thus lower the quantum yield in water (Yang et al., 2017). The introduction of shielding groups can partly reduce the intermolecular interaction caused by excessive conjugated system, thus improving the fluorescent brightness (Yang et al., 2018; Zhang et al., 2016). Compared with the D-A-D structures, the cyanine dyes have higher absorption coefficient and moderate quantum yield (Ding et al., 2019). Typically, cyanine dyes consist of two heterocyclic end groups connected by a polymethine chain with tunable lengths (Bricks et al., 2015). The π-conjugate strength can be enhanced by lengthening the polymethine chain or modifying heterocycle, effectively promoting the emission wavelength of cyanine dyes over 1,000 nm, such as IR-26, IR-1061, IR-1080 and Flav7 (Casalboni et al., 2003; Cosco et al., 2017; Li et al., 2018). However, there are still critical issues in developing a NIR-II dye for clinical trials, due to poor molecular stability and short circulation time.

In this Minireview, we summarized commercial NIR-II cyanine dyes with both NIR-II peak and off-peak emission (Antaris et al., 2017; Carr et al., 2018; Zhu et al., 2018a). Meanwhile, we describe recent developments in the synthesis of NIR-II cyanine derivatives, and outline the progress of the pharmacokinetics improvement and *in vivo* imaging applications of cyanine dyes.

## Synthesis of Near-Infrared-II Cyanine/Polymethine Dyes

### Commercial Cyanine Dyes With Both Peak and Tail NIR-II Emission

**FIGURE 1:**
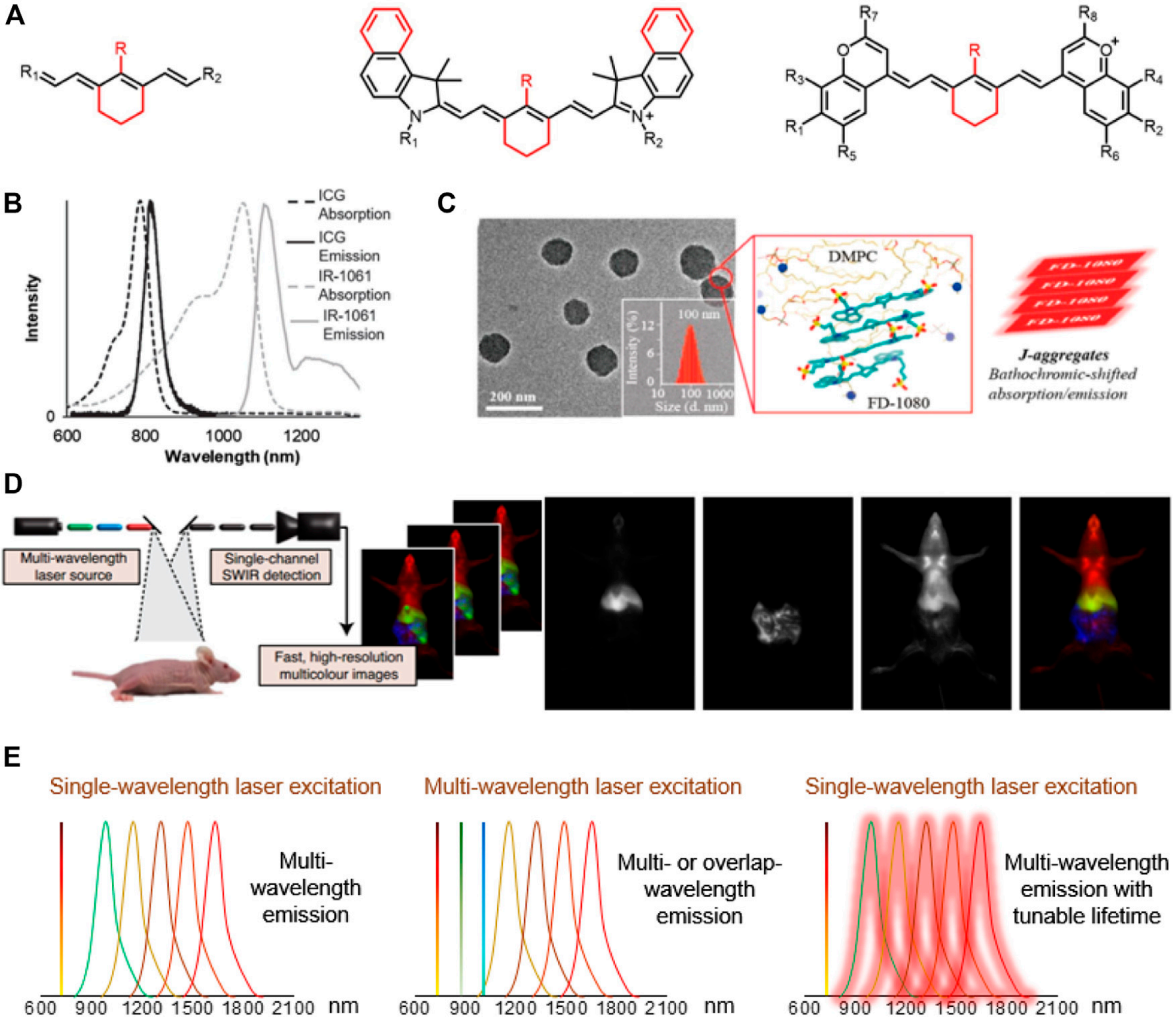
The development of NIR-II cyanine dyes for improving biological bioimaging, disease diagnosis and navigation surgery. **(A)** Core structures of NIR-I/II cyanine dyes. **(B)** Absorption and emission spectra of ICG and IR-1061 (in acetonitrile). Reproduced with permission from (Yeroslaysky et al., 2019). **(C)** TEM images, DLS, molecular dynamics simulation (red frame), and schematic diagram of FD-1080J-aggregates. Reproduced with permission from (Sun et al., 2019) Copyright 2019 ACS publications. **(D)** Multicolor imaging in mice using ICG, MeOFlav7 and JuloFlav7. Reproduced with permission from (Cosco et al., 2020) Copyright 2020 Springer Nature Publishing Group. **(E)** The diagram of multichannel biological imaging through multi-wavelength emission and tunable lifetime.

Small molecules with NIR-II emission have been synthesized and commercialized for years (Bricks et al., 2015), such as IR-26, IR-1061, IR-783 (Figure 1A). Some of them have been further expanded for imaging *in vivo*. A case in point is IR-1061, which was sequentially wrapped by polyacrylic acid (PAA) and DSPE-mPEG to form a stable nanocomplex in aqueous solution (Tao et al., 2013). The IR-1061 nanocomplex provided high-resolution vessel imaging in the 1,300–1,700 nm sub-NIR-II window, with a minimally discernible blood vessel width ∼150 μm. NIR-I dyes (ICG and IRDye800CW) spectroscopic mischaracterization on silicon detectors with insufficient NIR sensitivity falsely recorded their emission properties (Figure 1B). Recently, detecting on InGaAs camera have recovered the real emission spectrum of NIR-I-peak dyes. It has been found that most of NIR-I dyes have detectable NIR-II fluorescence (tail emission), and their NIR-II fluorescence intensity is even higher than that of some NIR-II peak emission dyes (Antaris et al., 2017; Zhu et al., 2018a). Highlighting the importance of NIR-II imaging with bright NIR-I-peak dyes that will fundamentally alter both clinical fluorescent imaging systems and current NIR-II dye synthetic strategies (Zhu et al., 2018b).

### Current Synthesis Strategies for NIR-II Peak Emission of Cyanine Dyes

Researchers have taken large efforts to design and synthesize NIR-II fluorophores with long wavelength, high quantum efficiency, good biocompatibility and optical/physiological stability, producing novel cyanine dye structures with improved optical properties (Figure 1A; Table 1). Zhang’s group designed a set of fluorophores with tunable wavelength emission named CXs by changing the number of methylene groups (Lei et al., 2019). Compared with commercial IR-26, CXs has better water solubility and optical stability. By increasing the quantity of methylene groups, the peak emission shifted from CX-1 at 920 nm, CX-2 at 1,032 nm to CX-3 at 1,140 nm. Inspired by IR-26 structure, Sletten’s group replaced the sulfur heteroatom in thiaflavylium to oxygen to enhance the fluorescence intensity (Cosco et al., 2017). The electron-donating dimethylamino group was added to compensate the conjugated system to ensure long-wave absorption/emission. They eventually synthesized a series of cyanine dyes by linking two dimethylamino flavylium heterocycles with a polymethylene chain (Flav7). They further found that the steric hindrance of substituents would affect the π-conjugation strength, so that the emission wavelength of Flav7 adjusted ∼80 nm through changing the position of the substituents (Pengshung et al., 2020). Zhang’s group also synthesized a NIR-II FD-1080 dye which can be excited at 1,064 nm (Li et al., 2018). Adding a cyclohexene group in the middle of the methylene chain effectively increased the stability of FD-1080. The water solubility of FD-1080 was significantly increased by introducing sulfonic acid groups on the heterocyclic ring and the quantum yield of FD-1080-FBS complex can be increased from 0.31 to 5.94%. In addition, the FD-1080J-aggregate was achieved through self-assembly with 1, 2-dimyristoyl-sn-glycero-3-phosphocholine (Figure 1C) (Sun et al., 2019). J-aggregate of FD-1080 has pushed the emission peaks to 1,370 nm, affording even clearer imaging contrast for vasculature visualization. With the benefit of FD-1080-J-aggregates labelled mesoporous implant, the researchers fabricated the MSTP-FDJ@PAA to guide osteosynthesis with minuscule invasion, high resolution, and real-time surgical navigation in the NIR-II bioimaging window (Sun et al., 2021). However, the lack of cyanine dyes with peak emission over 1,500 nm and precisely tunable-emission wavelength has unfortunately limited NIR-II imaging exploration.

**TABLE 1:** Typically reported peak-emission NIR-II fluorophores.

	λ_abs_/λ_em_	QY [%]	solvent	εφ[M^−1^cm^−1^]	Properties	Refs
FD 1080	1,043/1,089	0.44	DMSO	196	Water-soluble	Li et al. (2018)
Flav 7	1,026/1,045	0.53	DCM	1,250	Water-soluble by micelles	Cosco et al. (2017)
CX 3	1,089/1,135	0.082	DMSO	7	Water-soluble	Lei et al. (2019)
BTC 1070	1,012/1,066	0.04	DMSO	31	Water-soluble by micelles	Wang et al. (2019)
BTC 982	948/986	1.26	DMSO	2,520	Water-soluble by micelles	Wang et al. (2019)
LZ 1055	1,058/1,100	3.89	DMSO	5,700	Water-soluble	Li et al. (2020)
IR 1061	1,061/1,100	0.59	DCM	1,400	Water-soluble by micelles	Tao et al. (2013)

### The Enhancement of NIR-II Brightness

Generally, the quantum yield of a single NIR-II fluorescent molecule, owing to the small HOMO\LUMO energy gap and excessively large conjugated system lead to low structural rigidity (Lei et al., 2021), is relatively low. The intramolecular twist (Lei et al., 2021), the interaction between molecules or the interaction between molecules and water will also reduce the brightness in biological conditions. Therefore, researchers sought to increase the intermolecular distance by increasing the steric hindrance between molecules to increase the quantum yield of NIR-II dyes (Li et al., 2018). The Forster resonance energy transfer (FRET) effect between CXs can effectively enhance the brightness of CX-3, thereby realizing the response to biomarker of drug-induced hepatotoxicity OONO^−^ (Lei et al., 2019). The complex formed by the protein (albumin) and the dye (NIR-I-peak cyanine) can also effectively increase the brightness by restricting the intramolecular rotation of the fluorophore (Tian et al., 2019). Besides, recent academic publication has shown that in addition to the length of the polymethine chain, substituents affect the brightness of cyanine dyes (Cosco et al., 2017; Cosco et al., 2020; Lei et al., 2019; Pengshung et al., 2020; Wang et al., 2019). For example, Sletten’s group found choosing substituents with fewer vibration modes could significantly increase the fluorescence quantum yield of the dye, which is attributed to the reduction of non-radiative energy dissipation (Cosco et al., 2021).

## Pharmacokinetics Improvement and *In Vivo* Imaging of NIR-II Cyanine Dyes

Although NIR-II dye have not been approved for clinical use, small animal experiments have repeatedly shown that NIR-II-peak dyes and NIR-I-peak dyes with NIR-II tail emission has commendable performance including real-time vascular imaging and tumor recognition. To address the short-blood-circulation issue of current NIR-I-peak dyes, bovine serum albumin (BSA) was used to self-assemble with cyanine dyes (e.g., IR-783) into IR-783@BSA complex, which efficiently prolonged NIR-I/II bioimaging window (Tian et al., 2019). A NIR-II-peak dye (LZ-1105) was also developed to specifically bond with fibrinogen, providing the long blood circulation time for real-time angiography (Li et al., 2020). In addition, an anti-quenched fluorophore BTC1070 was synthesized with response to pH through the process of nitrogen protonation/deprotonation, realizing non-invasive and accurate ratiometric imaging of gastric pH *in vivo* (Wang et al., 2019). Sletten’s group further rationally changed the substituents of Flav7 to screen two dyes MeOFlav7 and JuloFlav7, which can match the 980 and 1,064 nm commercial lasers, respectively. By coupling with ICG as the third channel, they finally achieved three-color, high-speed and real-time bioimaging (Figure 1D,E) (Cosco et al., 2020). With the added companion of chromenylium dyes to flavylium dyes, they performed the four-channel bioimaging (ICG, JuloChrom5, Chrom7 and JuloFlav7) (Cosco et al., 2021).

## Perspective and Challenges

NIR-II organic dyes are relatively facile to synthesize, easily modifiable, and have low biological toxicity, providing great potential for clinical translation. ICG and IRDye800CW are by far the most critical dyes to display NIR-II emission given their widespread use in modern medical imaging (Hu et al., 2020). In particular, the cyanine NIR-II fluorophore can effectively optimize its spectroscopic properties by changing the methylene groups and heterocyclic donor, thus overcoming the color-barrier for multichannel biological imaging and improving bioimaging quality to the longer NIR-II wavelength regions (Zhu et al., 2019). In addition to improving the molecular structure, researchers also tried to increase the steric hindrance or designed FRET-based fluorescent probes, effectively increasing the emission wavelength and the quantum yield (Lei et al., 2019). Imaging in the longer NIR-II sub-window (i.e. 1,500–1,700 nm NIR-IIb) provides nealy “zero” background (reduced photon scattering/autofluorescence and water absorbance) and even better imaging quality, however, organic dyes with a maximum emission wavelength beyond 1,200 and/or 1,500 nm are still rare. This requires the design of a larger conjugated system, and a more optimized molecular structure of the dye molecules. All in all, strategies to improve the photophysical properties, pharmacokinetics and *in vivo* imaging of NIR-II cyanine dyes still remain to be explored.

## References

[B1] Antaris A. L. Chen H. Cheng K. Sun Y. Hong G. Qu C. (2016). A Small-Molecule Dye for NIR-II Imaging. Nat. Mater 15, 235–242. 10.1038/nmat4476 26595119

[B2] Antaris A. L. Chen H. Diao S. Ma Z. Zhang Z. Zhu S. (2017). A High Quantum Yield Molecule-Protein Complex Fluorophore for Near-Infrared II Imaging. Nat. Commun. 8, 15269. 10.1038/ncomms15269 28524850 PMC5454457

[B3] Bricks J. L. Kachkovskii A. D. Slominskii Y. L. Gerasov A. O. Popov S. V. (2015). Molecular Design of Near Infrared Polymethine Dyes: A Review. Dyes Pigm. 121, 238–255. 10.1016/j.dyepig.2015.05.016

[B4] Bruns O. T. Bischof T. S. Harris D. K. Franke D. Shi Y. Riedemann L. (2017). Next-generation *In Vivo* Optical Imaging with Short-Wave Infrared Quantum Dots. Nat. Biomed. Eng. 1, 0056. 10.1038/s41551-017-0056 29119058 PMC5673283

[B5] Carr J. A. Franke D. Caram J. R. Perkinson C. F. Saif M. Askoxylakis V. (2018). Shortwave Infrared Fluorescence Imaging with the Clinically Approved Near-Infrared Dye Indocyanine green. Proc. Natl. Acad. Sci. USA 115, 4465–4470. 10.1073/pnas.1718917115 29626132 PMC5924901

[B6] Casalboni M. De Matteis F. Prosposito P. Quatela A. Sarcinelli F. (2003). Fluorescence Efficiency of Four Infrared Polymethine Dyes. Chem. Phys. Lett. 373, 372–378. 10.1016/s0009-2614(03)00608-0

[B7] Chen W. Cheng C.-A. Cosco E. D. Ramakrishnan S. Lingg J. G. P. Bruns O. T. (2019). Shortwave Infrared Imaging with J-Aggregates Stabilized in Hollow Mesoporous Silica Nanoparticles. J. Am. Chem. Soc. 141, 12475–12480. 10.1021/jacs.9b05195 31353894 PMC6746239

[B8] Cosco E. D. Arús B. A. Spearman A. L. Atallah T. L. Lim I. Leland O. S. (2021). Bright Chromenylium Polymethine Dyes Enable Fast, Four-Color *In Vivo* Imaging with Shortwave Infrared Detection. J. Am. Chem. Soc. 143, 6836–6846. 10.1021/jacs.0c11599 33939921 PMC8327756

[B9] Cosco E. D. Caram J. R. Bruns O. T. Franke D. Day R. A. Farr E. P. (2017). Flavylium Polymethine Fluorophores for Near‐ and Shortwave Infrared Imaging. Angew. Chem. Int. Ed. 56, 13126–13129. 10.1002/anie.201706974 28806473

[B10] Cosco E. D. Spearman A. L. Ramakrishnan S. Lingg J. G. P. Saccomano M. Pengshung M. (2020). Shortwave Infrared Polymethine Fluorophores Matched to Excitation Lasers Enable Non-invasive, Multicolour *In Vivo* Imaging in Real Time. Nat. Chem. 12, 1123–1130. 10.1038/s41557-020-00554-5 33077925 PMC7680456

[B11] Ding F. Fan Y. Sun Y. Zhang F. (2019). Beyond 1000 Nm Emission Wavelength: Recent Advances in Organic and Inorganic Emitters for Deep‐Tissue Molecular Imaging. Adv. Healthc. Mater. 8, 1900260. 10.1002/adhm.201900260 30983165

[B12] Fan Y. Wang P. Lu Y. Wang R. Zhou L. Zheng X. (2018). Lifetime-engineered NIR-II Nanoparticles Unlock Multiplexed *In Vivo* Imaging. Nat. Nanotech 13, 941–946. 10.1038/s41565-018-0221-0 30082923

[B13] Frangioni J. (2003). *In Vivo* near-infrared Fluorescence Imaging. Curr. Opin. Chem. Biol. 7, 626–634. 10.1016/j.cbpa.2003.08.007 14580568

[B14] Hong G. Antaris A. L. Dai H. (2017). Near-infrared Fluorophores for Biomedical Imaging. Nat. Biomed. Eng. 1. 10.1038/s41551-016-0010

[B15] Hong G. Diao S. Chang J. Antaris A. L. Chen C. Zhang B. (2014). Through-skull Fluorescence Imaging of the Brain in a New Near-Infrared Window. Nat. Photon 8, 723–730. 10.1038/nphoton.2014.166 PMC5026222 27642366

[B16] Hu Z. Fang C. Li B. Zhang Z. Cao C. Cai M. (2020). First-in-human Liver-Tumour Surgery Guided by Multispectral Fluorescence Imaging in the Visible and Near-Infrared-I/II Windows. Nat. Biomed. Eng. 4, 259–271. 10.1038/s41551-019-0494-0 31873212

[B17] Lei Z. Sun C. Pei P. Wang S. Li D. Zhang X. (2019). Stable, Wavelength‐Tunable Fluorescent Dyes in the NIR‐II Region for *In Vivo* High‐Contrast Bioimaging and Multiplexed Biosensing. Angew. Chem. Int. Ed. 58, 8166–8171. 10.1002/anie.201904182 31008552

[B18] Lei Z. Zhang F. (2021). Molecular Engineering of NIR‐II Fluorophores for Improved Biomedical Detection. Angew. Chem. Int. Ed. 60, 16294–16308. 10.1002/anie.202007040 32780466

[B19] Li B. Lu L. Zhao M. Lei Z. Zhang F. (2018). An Efficient 1064 Nm NIR-II Excitation Fluorescent Molecular Dye for Deep-Tissue High-Resolution Dynamic Bioimaging. Angew. Chem. Int. Ed. 57, 7483–7487. 10.1002/anie.201801226 29493057

[B20] Li B. Zhao M. Feng L. Dou C. Ding S. Zhou G. (2020). Organic NIR-II Molecule with Long Blood Half-Life for *In Vivo* Dynamic Vascular Imaging. Nat. Commun. 11, 3102. 10.1038/s41467-020-16924-z 32555157 PMC7303218

[B21] Li C. Li F. Zhang Y. Zhang W. Zhang X.-E. Wang Q. (2015). Real-Time Monitoring Surface Chemistry-dependent *In Vivo* Behaviors of Protein Nanocages via Encapsulating an NIR-II Ag2S Quantum Dot. ACS Nano 9, 12255–12263. 10.1021/acsnano.5b05503 26496067

[B22] Liu H. Hong G. Luo Z. Chen J. Chang J. Gong M. (2019). Atomic‐Precision Gold Clusters for NIR‐II Imaging. Adv. Mater. 31, 1901015. 10.1002/adma.201901015 31576632

[B23] Loynachan C. N. Soleimany A. P. Dudani J. S. Lin Y. Najer A. Bekdemir A. (2019). Renal Clearable Catalytic Gold Nanoclusters for *In Vivo* Disease Monitoring. Nat. Nanotechnol. 14, 883–890. 10.1038/s41565-019-0527-6 31477801 PMC7045344

[B24] Naczynski D. J. Tan M. C. Zevon M. Wall B. Kohl J. Kulesa A. (2013). Rare-earth-doped Biological Composites as *In Vivo* Shortwave Infrared Reporters. Nat. Commun. 4, 2199. 10.1038/ncomms3199 23873342 PMC3736359

[B25] Pengshung M. Li J. Mukadum F. Lopez S. A. Sletten E. M. (2020). Photophysical Tuning of Shortwave Infrared Flavylium Heptamethine Dyes via Substituent Placement. Org. Lett. 22, 6150–6154. 10.1021/acs.orglett.0c02213 32790432 PMC7542986

[B26] Sun C. Li B. Zhao M. Wang S. Lei Z. Lu L. (2019). J-aggregates of Cyanine Dye for NIR-II *In Vivo* Dynamic Vascular Imaging beyond 1500 Nm. J. Am. Chem. Soc. 141, 19221–19225. 10.1021/jacs.9b10043 31746598

[B27] Sun C. Sun X. Pei P. He H. Ming J. Liu X. (2021). NIR‐II J‐Aggregates Labelled Mesoporous Implant for Imaging‐Guided Osteosynthesis with Minimal Invasion. Adv. Funct. Mater. 31, 2100656. 10.1002/adfm.202100656

[B28] Tao Z. Hong G. Shinji C. Chen C. Diao S. Antaris A. L. (2013). Biological Imaging Using Nanoparticles of Small Organic Molecules with Fluorescence Emission at Wavelengths Longer Than 1000 Nm. Angew. Chem. Int. Ed. 52, 13002–13006. 10.1002/anie.201307346 24174264

[B29] Tian R. Zeng Q. Zhu S. Lau J. Chandra S. Ertsey R. (2019). Albumin-chaperoned Cyanine Dye Yields Superbright NIR-II Fluorophore with Enhanced Pharmacokinetics. Sci. Adv. 5, eaaw0672. 10.1126/sciadv.aaw0672 31548981 PMC6744268

[B30] Wang S. Fan Y. Li D. Sun C. Lei Z. Lu L. (2019). Anti-quenching NIR-II Molecular Fluorophores for *In Vivo* High-Contrast Imaging and pH Sensing. Nat. Commun. 10, 1058. 10.1038/s41467-019-09043-x 30837470 PMC6401027

[B31] Welsher K. Liu Z. Sherlock S. P. Robinson J. T. Chen Z. Daranciang D. (2009). A Route to Brightly Fluorescent Carbon Nanotubes for Near-Infrared Imaging in Mice. Nat. Nanotech 4, 773–780. 10.1038/nnano.2009.294 PMC2834239 19893526

[B32] Yang Q. Hu Z. Zhu S. Ma R. Ma H. Ma Z. (2018). Donor Engineering for NIR-II Molecular Fluorophores with Enhanced Fluorescent Performance. J. Am. Chem. Soc. 140, 1715–1724. 10.1021/jacs.7b10334 29337545

[B33] Yang Q. Ma Z. Wang H. Zhou B. Zhu S. Zhong Y. (2017). Rational Design of Molecular Fluorophores for Biological Imaging in the NIR-II Window. Adv. Mater. 29, 1605497. 10.1002/adma.201605497 28117499

[B34] Yeroslaysky G. Umezawa M. Okubo K. Nigoghossian K. Doan Thi Kim D. Kamimura M. (2019). Photostabilization of Indocyanine Green Dye by Energy Transfer in Phospholipid-PEG Micelles. J. Photopolymer Sci. Technology 32, 115–121. 10.2494/photopolymer.32.115

[B35] Zhang X.-D. Wang H. Antaris A. L. Li L. Diao S. Ma R. (2016). Traumatic Brain Injury Imaging in the Second Near-Infrared Window with a Molecular Fluorophore. Adv. Mater. 28, 6872–6879. 10.1002/adma.201600706 27253071 PMC5293734

[B36] Zhang Y. Zhang Y. Hong G. He W. Zhou K. Yang K. (2013). Biodistribution, Pharmacokinetics and Toxicology of Ag2S Near-Infrared Quantum Dots in Mice. Biomaterials 34, 3639–3646. 10.1016/j.biomaterials.2013.01.089 23415643

[B37] Zhong Y. Ma Z. Zhu S. Yue J. Zhang M. Antaris A. L. (2017). Boosting the Down-Shifting Luminescence of Rare-Earth Nanocrystals for Biological Imaging beyond 1500 Nm. Nat. Commun. 8, 737. 10.1038/s41467-017-00917-6 28963467 PMC5622117

[B38] Zhu S. Chen X. (2019). Overcoming the Colour Barrier. Nat. Photon. 13, 515–516. 10.1038/s41566-019-0500-9

[B39] Zhu S. Hu Z. Tian R. Yung B. C. Yang Q. Zhao S. (2018a). Repurposing Cyanine NIR-I Dyes Accelerates Clinical Translation of Near-Infrared-II (NIR-II) Bioimaging. Adv. Mater. 30, 1802546. 10.1002/adma.201802546 29985542

[B40] Zhu S. Yung B. C. Chandra S. Niu G. Antaris A. L. Chen X. (2018b). Near-Infrared-II (NIR-II) Bioimaging via Off-Peak NIR-I Fluorescence Emission. Theranostics 8, 4141–4151. 10.7150/thno.27995 30128042 PMC6096392

